# Alleviation of an Arctic Sea Ice Bias in a Coupled Model Through Modifications in the Subgrid‐Scale Orographic Parameterization

**DOI:** 10.1029/2020MS002111

**Published:** 2020-09-21

**Authors:** Guillaume Gastineau, François Lott, Juliette Mignot, Frederic Hourdin

**Affiliations:** ^1^ UMR LOCEAN, Sorbonne Université/IRD/MNHN/CNRS, IPSL Paris France; ^2^ UMR LMD, ENS/Sorbonne Université/CNRS/Ecole Polytechnique, IPSL Paris France

**Keywords:** climate models, orography, atmospheric dynamics, air‐sea interaction, Arctic Ocean

## Abstract

In climate models, the subgrid‐scale orography (SSO) parameterization imposes a blocked flow drag at low levels that is opposed to the local flow. In IPSL‐CM6A‐LR, an SSO lift force is also applied perpendicular to the local flow to account for the effect of locally blocked air in narrow valleys. Using IPSL‐CM6A‐LR sensitivity experiments, it is found that the tuning of both effects strongly impacts the atmospheric circulation. Increasing the blocking and reducing the lift lead to an equatorward shift of the Northern Hemisphere subtropical jet and a reduction of the midlatitude eddy‐driven jet speed. It also improves the simulated synoptic variability, with a reduced storm‐track intensity and increased blocking frequency over Greenland and Scandinavia. Additionally, it cools the polar lower troposphere in boreal winter. Transformed Eulerian Mean diagnostics also show that the low‐level eddy‐driven subsidence over the polar region is reduced consistent with the simulated cooling. The changes are amplified in coupled experiments when compared to atmosphere‐only experiments, as the low‐troposphere polar cooling is further amplified by the temperature and albedo feedbacks resulting from the Arctic sea ice growth. In IPSL‐CM6A‐LR, this corrects the warm winter bias and the lack of sea ice that were present over the Arctic before adjusting the SSO parameters. Our results, therefore, suggest that the adjustment of SSO parameterization alleviates the Arctic sea ice bias in this case. However, the atmospheric changes induced by the parametrized SSO also impact the ocean, with an equatorward shift of the Northern Hemisphere oceanic gyres and a weaker Atlantic meridional overturning circulation.

## Key Points

- The adjustment of the parametrized orography in climate models can alleviate the near‐surface winter biases over the Arctic
- Increasing low‐level drag reduces Northern Hemisphere stationary wave amplitudes and shifts the subtropical jet equatorward
- Increasing low‐level drag increases the Arctic sea ice coverage and reduces the Atlantic meridional overturning circulation

## 1 Introduction

The representation of subgrid‐scale orography (SSO) in global climate models is still considered to be a major challenge (Sandu et al., 2019). Although the large‐scale orography influence is partly resolved in standard resolution models (~100 km), processes like gravity waves, the blocking effect of small‐scale mountains and hills, and the associated turbulence indeed require the use of very high resolution models (<1 km). Most global climate models use SSO parameterizations to capture the missing effect of orographic gravity waves and low‐level blocking (Lott & Miller, 1997; Palmer et al., 1986). Although early parameterizations only included mountain wave drags at upper levels that are oriented against the low‐level flow, more recent schemes start to take into account directional effects (Bacmeister, 1993; Baines & Palmer, 1985; Garner, 2005). In such schemes, the gravity wave drag is in an intermediate direction between the low‐level winds and the minor axis of the SSO ridges, depending on the degree of anisotropy. Progress was also made in the late 1990s, with the inclusion of low‐level blocked flow drag. In most schemes, its intensity is also a function of anisotropy, but its direction is often assumed to be opposed to the low‐level winds (Lott & Miller, 1997). Although the inclusion of directional effects was never thoroughly tested for the gravity waves in the aforementioned studies, it soon appeared that applying low‐level drag alone was not sufficient to improve the simulated stationary planetary waves (Lott, 1999). Lott (1999) then proposed to implement additionally the effect of lift forces perpendicular to the local flow. Based on an analogy with the effect of the envelope orography (Wallace et al., 1983), the lift force represents the dynamical separation of the air in narrow valleys from the large‐scale flow. To some extent, it demonstrates that direction matters: The component of the forces perpendicular to the winds does not decelerate the flow directly, but it still distorts it efficiently when applied regionally. The lift force mimics the vortex stretching effect over large‐scale mountains and yields a realistic planetary wave with little zonal‐mean flow deceleration (Lott, 1999). As model resolution increases, one could have expected that these issues become less critical. Yet it happens not to be the case. The spectrum of unresolved and resolved processes is currently still not well understood, and much care is needed to evaluate the influence of parameterized orography (van Niekerk et al., 2016). For instance, Zadra (2015) found that the parametrized surface stress is highly model dependent, with impacts at all time scales.

Furthermore, how the ocean is impacted by SSO parameterizations remains not well understood. Based on the similarity between Climate Model Intercomparison Project Phase 5 (CMIP5) model midlatitude biases and changes simulated while suppressing SSO effects, Pithan et al. (2016) suggested that much of the CMIP5 climate model biases could be alleviated by increasing the parametrized drag. van Niekerk et al. (2017) also found that the CMIP5 model biases in the position of the North Atlantic and North Pacific jets found can be linked to the parametrized low‐level drag. Another relevant example of the impact of SSO parametrizations concerns the tuning of the GFDL model where different SSO schemes were tested (Zhao et al., 2018). It was noted that increasing orographic drag was associated with a cooling of Arctic surface air temperature. However, the physics and feedbacks related to air‐sea coupling behind these corrections need to be analyzed according to Held et al. (2019). Such analysis is indeed essential: since mountain wave drags are often introduced to reduce cold biases (Palmer et al., 1986) through downward control, we have to understand how low‐level parametrized drag can result in opposite effects.

Following on from these studies, we investigate here the effect of SSO parameterization in the Arctic region. The intention is also to reduce a warm winter bias in the lower troposphere over the Arctic sea ice. Such bias, previously linked to the poor simulation of the planetary boundary layer (Tjernström & Graversen, 2009) or clouds (Walsh et al., 2009), is indeed present in many models (Graham et al., 2019).

The present study addresses these issues with the IPSL‐CM6A‐LR model (Boucher et al., 2020), with a focus on its atmospheric component, LMDZ6A (Hourdin et al., 2020). The IPSL‐CM6A‐LR model was used to perform the Coupled Model Intercomparison Project Phase 6 (CMIP6; Eyring et al., 2016) simulations. The study was motivated by a difficulty encountered during the tuning of this model configuration, namely, a systematic underestimation of the Arctic sea ice at the end of winter. This deficiency was in part attributed to a bad representation of the stationary planetary waves. In our case, this produces an overestimation of warm air advection from low latitudes to the Arctic in winter, thereby inhibiting winter sea ice growth. This motivated a tuning of the SSO parameterization, which indeed appeared to play a crucial role in the representation of Arctic sea ice. The simulations presented in this paper reassess this particular tuning step through sensitivity experiments starting from the final version of the model, using the atmospheric model component LMDZ6A both in stand‐alone atmospheric mode and coupled to the ocean. Another goal of this study is to assess the performance of IPSL‐CM6A‐LR regarding Northern Hemisphere climate characteristics, as the CMIP6 simulations produced by IPSL‐CM6A‐LR will be used next in many studies. We will explore the sensitivity of this model to the SSO drag and lift effect, and we will illustrate why and how the Arctic and midlatitude climate is modified by adjusting both effects.

This manuscript is organized as follows: The model and the methodology are presented in section 2. Sensitivity atmosphere‐only experiments are analyzed in section 3, and coupled ones in section 4. Conclusions are given in section 5.

## 2 Methods

### 2.1 Atmosphere‐Only Experiments

This study uses the land‐atmosphere components of the IPSL‐CM6A‐LR model used for CMIP6, called LMDZOR6, in stand‐alone mode, forced by sea surface temperature (SST) and sea ice concentration. LMDZOR6 is based on the atmospheric model LMDZ version 6, which is described in a companion paper of the same Special Collection (Hourdin et al., 2020). It has a resolution of 2.5° × 1.25° and 79 vertical levels that extend up to 80 km (~1.5 Pa). It is coupled to the ORCHIDEE (Boucher et al., 2020) land surface model. In LMDZ6, the convective and planetary boundary layer scheme was revisited (Hourdin et al., 2020). A refinement of the vertical grid and a new adjustment of the thresholds of stability functions were implemented for a better representation of the very stable atmospheric boundary layer (Vignon et al., 2017). The scheme producing SSO gravity waves drag is also used to produce a shear production term in the prognostic turbulent kinetic equation of the planetary boundary layer scheme. This produces a turbulent orographic form drag, which was carefully validated over the Antarctica ice sheet (see details in the appendix of Cheruy et al., 2020). In LMDZ, the SSO parameterization applies gravity wave drag at upper levels and low‐level drag and lift forces at the model levels that intersect the SSO. The low‐level drag force represents the blocking effect of orography. It is opposed to the local wind (Lott & Miller, 1997). The lift represents the effect of blocked air in narrow valleys intensifying the vortex compression (Lott, 1999). Among others, the low‐level drag and lift effects depend on *C*_*d*_ and *C*_*l*_, respectively, which are two dimensionless scaling parameters that need to be carefully adjusted. *C*_*d*_ directly controls the blocked flow component of the drag, while *C*_*l*_ controls the amplitude of the lift force.

We integrate several LMDZOR6 simulations (see Table 1) using a repeated annual cycle of SST and sea ice concentration as boundary conditions, calculated from a climatology of the 1979–2008 forcings designed for the CMIP6 Atmospheric Model Intercomparison Project (AMIP; Durack & Taylor, 2018). The simulations are performed over 30 yr, with fixed present‐day external forcings, using the CMIP6 (Eyring et al., 2016) plant functional type maps, greenhouse gases, ozone, aerosols, and solar forcing of the year 2000. The control simulation, referred to as Atm‐6A, uses the standard value for *C*_*d*_ and *C*_*l*_ from the IPSL‐CM6A‐LR CMIP6 configuration. We also use the ensemble of 10 AMIP simulations produced for CMIP6 with the same atmospheric model (Boucher et al., 2020). These simulations are identical to Atm‐6A, but they used interannual SST, sea ice, and external forcings. We also focus on the 1979–2008 period in this ensemble.

**Table 1:** Presentation of the Main Simulation Discussed in This Study

Name	Model	Members	Length (in yr)	Parameters	
				*C*_*d*_	*C*_*l*_
Atm‐6A	LMDZOR‐6A	1	30	0.6	0.1
Atm‐5DL	LMDZOR‐6A	1	30	0.2	0.25
Atm‐6A‐Drg+	LMDZOR‐6A	1	30	1.2	0.1
Atm‐6A‐Drg−	LMDZOR‐6A	1	30	0.2	0.1
Atm‐6A‐Lft−	LMDZOR‐6A	1	30	0.6	0.0
Atm‐6A‐Lft+	LMDZOR‐6A	1	30	0.6	1.0
AO‐6A	IPSL‐CM6A‐LR	1	200	0.6	0.1
AO‐5DL	IPSL‐CM6A‐LR	5	80	0.2	0.25

We also integrate simulations identical to Atm‐6A, but using increased (decreased) values of *C*_*d*_ in Atm‐6A‐Drg+ (Atm‐6A‐Drg−) and similarly for *C*_*l*_ in Atm‐6A‐Lft+ (Atm‐6A‐Lft−). The exact values are given in Table 1. In case *C*_*l*_ is reduced to 0, the orographic lift parametrization is deactivated. Lastly, a simulation combining these two changes with an increased *C*_*d*_ and a decreased *C*_*l*_ is referred to as Atm‐5DL. This corresponds to the setup of the previous version of the atmospheric model, named LMDZ5A (Hourdin et al., 2006) and used for CMIP5.

Hereafter, the significance level for the difference of any variable between two simulations is given by the *p*‐value of a Student's *t* test assuming equal variances. The number of degrees of freedom used in the *t* tests is *n* − 2, with *n* the number of years or seasons considered for computing the average value.

### 2.2 Coupled Experiments

We also use the IPSL‐CM6A‐LR (Boucher et al., 2020) atmosphere‐ocean general circulation model (AOGCM), which consists of LMDZOR6 coupled to the NEMO ocean model using a nominal horizontal resolution of about 1° with refinement at the equator and poles (eORCA1 grid), 75 vertical levels, and the LIM3 sea ice module. The Northern Hemisphere climate of the preindustrial CMIP6 control simulation of this model shows a marked centennial variability linked to Atlantic meridional overturning circulation (AMOC) fluctuations (Boucher et al., 2020). This variability is also visible in CMIP6 historical simulations. This motivates the use of a 200‐yr period of the preindustrial simulation as a control for our sensitivity study, to ensure that this variability does not affect our results. We arbitrarily chose to focus here on the 1990–2189 model years. This simulation is referred to as AO‐6A. Although preindustrial external forcings are quite different from present‐day ones, the results presented next are likely unchanged in present‐day conditions.

Starting from this preindustrial configuration, we integrate a five‐member ensemble, called AO‐5DL, using the values of *C*_*d*_ and *C*_*l*_ from the previous CMIP5 IPSL model version (increased *C*_*d*_ and decreased *C*_*l*_, as previously described, see Table 1). The setup is otherwise identical to AO‐6A. The members last 80 yr and start at dates sampled every 40 yr in the given 200‐yr period. The first 30‐yr period of each ensemble is discarded. The integration of such ensemble ensures an accurate estimation of the SSO influence so that the important centennial variability present in IPSL‐CM6A‐LR does not affect too much the results.

### 2.3 Observations

Monthly and/or daily sea ice concentration, sea level pressure (SLP), geopotential height, air temperature, and zonal and meridional wind are retrieved from the ERA‐Interim reanalysis interpolated onto a 2° grid (Dee et al., 2011) over the 1979–2014 period.

## 3 Impacts on the Atmospheric Circulation

### 3.1 Mean State

**Figure 1:**
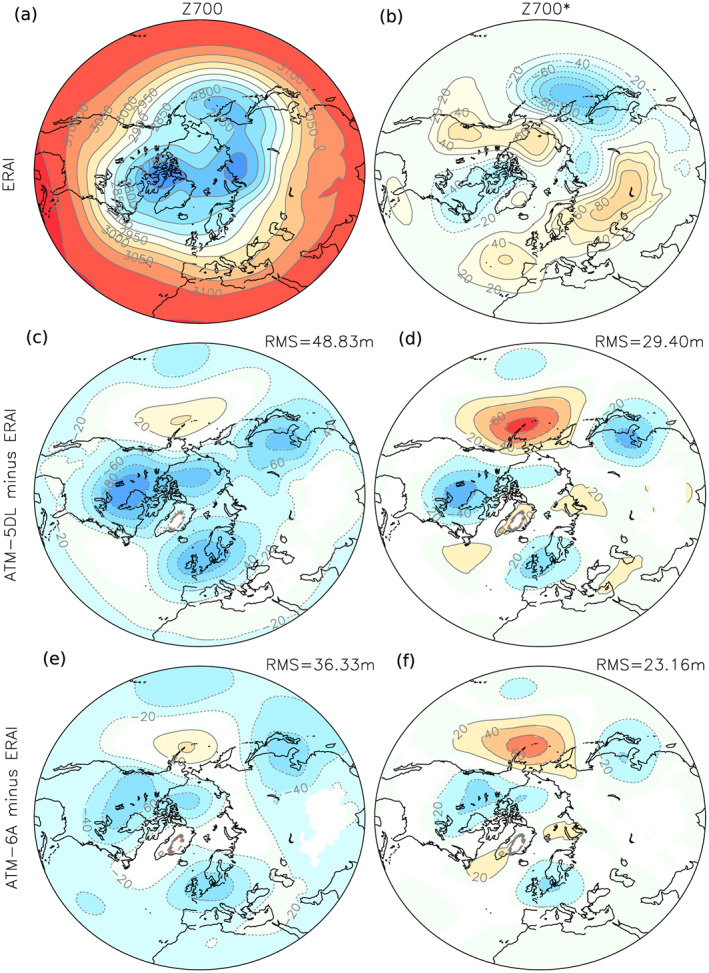
(a) Geopotential height at 700 hPa averaged over the winter months (DJFM) in ERA‐Interim (1979–2014) and (b) its zonally asymmetric component. (c) and (d) are the same as (a) and (b), but for the difference Atm‐5DL minus ERA‐Interim. (e) and (f) are the same as (a) and (b), but for the difference Atm‐6A minus ERA‐Interim. In (c)–(f) panels, the root mean square (RMS) of the difference over 20–90°N is also given on top of each panel; only grid points with statistical significance lower than 10% are colored.

The influence of the SSO parameters on the Arctic climate is first assessed in atmosphere‐only experiments. Although the atmospheric component of LMDZOR6 includes a series of physical updates as compared to previous versions (see the previous section), stationary planetary wave errors over Northern America and Northern Atlantic remain when using the SSO parameters of the CMIP5 version. More specifically, the stationary planetary wave is much more pronounced than in reanalysis, with the three troughs visible in the 700‐hPa geopotential height, located over North America, western Europe, and eastern Asia being deeper than in ERA‐Interim (Figure 1c). This can result in meridional exchanges, for instance, from enhanced (reduced) advection of warm air from the midlatitudes to the polar regions where most of the Arctic sea ice forms in winter. The zonally asymmetric changes (Figure 1d) also show that the stationary wave is shifted west when compared to ERA‐Interim over eastern Asia, North Pacific, and North America.

The overestimated stationary wave amplitude might be corrected by imposing more orographic drag and, therefore, decelerating the flow (Sandu et al., 2016). For instance, such an effect of increasing low‐level drag was found by van Niekerk et al. (2017), although opposed changes were found north of 60°N. Furthermore, as the lift force leads to more vortex stretching over large‐scale mountains, reducing the lift effect may also reduce the planetary wave with little impacts on the zonal flow, as discussed in Lott (1999). We, therefore, chose to reduce *C*_*l*_ (from 0.25 to 0.1) and increase *C*_*d*_ (from 0.2 to 0.6) between Atm‐5DL and Atm‐6A (see Table 1). Figures 1e and 1f show that doing so, the errors on both the planetary waves and the zonally symmetric part of the low‐level jet are reduced. The improvement is quantified in Figure 1 by the root mean square error (RMSE) in the 20–90°N latitude band, which is reduced for both the 700‐hPa geopotential height (from 48.8 to 36.3 m) and its asymmetric component (from 29.4 to 23.2 m).

**Figure 2:**
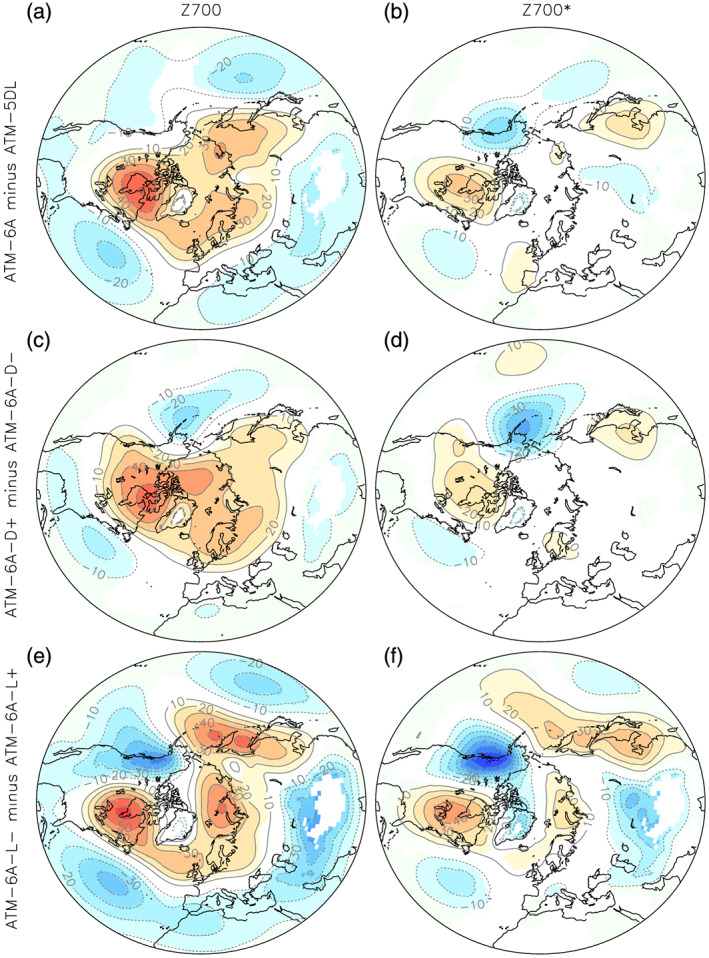
Difference of the simulated DJFM (a) 700‐hPa geopotential height and (b) its zonally asymmetric component, in Atm‐6A minus Atm‐5DL. (c) and (d) are the same as (a) and (b), but for Atm‐6A‐Drg+ minus Atm‐6A‐Drg−. (e) and (f) are the same as (a) and (b), but for Atm‐6A‐Lft− minus Atm‐6A‐Lft+. Only grid points with statistical significance lower than 10% are colored.

To illustrate how the lift and drag can be combined to modify the planetary wave and the zonal‐mean flow, Figure 2 shows the differences between Atm‐6A and Atm‐5DL (Figures 2a and 2b) as well as the difference between runs where the drag is enhanced by a factor of 6 (*C*_*d*_ increased from 0.2 to 1.2; Figures 2c and 2d) and differences between a run with strong lift and a run with no lift (*C*_*l*_ parameter decreased from 1.0 to 0.0; Figures 2e and 2f). In these sensitivity simulations, we see that the drag alone can well decelerate the global flow (cf. Figures 2a and 2c), with a weakening of the tropospheric polar jet. This effect of the SSO on the zonal‐mean flow is consistent with the effect expected from mountain drags onto the zonal‐mean atmospheric mass distribution (Lott & D'andrea, 2005; Lott et al., 2004) and is consistent with the results of previous studies (Sandu et al., 2016; Zadra et al., 2003). The meridional pressure gradient produced is consistent with an anomalous geostrophic westward zonal flow due to the low‐level blocking. The drag also reduces the trough over north‐eastern America and tends to produce a strong ridge to the west of the Alaska peninsula. The lift force is less efficient in producing an axisymmetric response (cf. Figures 2c and 2e) but much more efficient in producing a planetary wave (Figures 2d and 2f).

Lastly, we note that the influence of varying SST does not change the overall standing planetary wave pattern. Indeed, the ensemble mean of AMIP CMIP6 experiments using interannual forcings shows 700‐hPa geopotential height asymmetries largely similar to the simulation Atm‐6A using climatological surface boundary conditions (see Supporting Information Figure S1).

### 3.2 Atmospheric Variability

**Figure 3:**
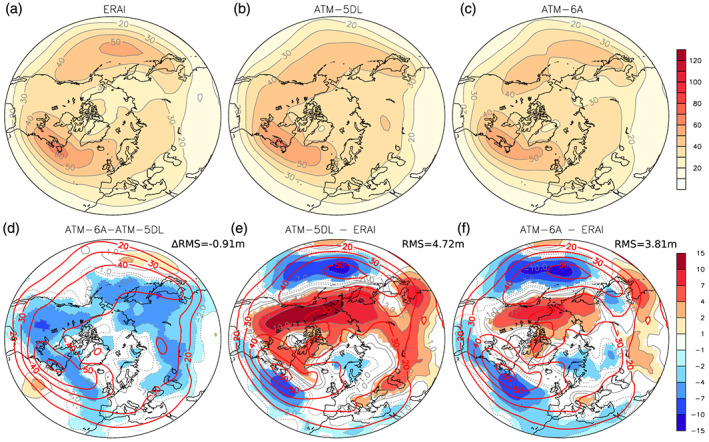
Daily band‐pass (2.5–6 days) DJFM 500‐hPa geopotential height standard deviation, in m, for (a) ERA‐Interim, (b) Atm‐5DL, (c) Atm‐6A, (d) Atm‐6A minus Atm‐5DL, (e) Atm‐5DL minus ERA‐Interim, and (f) Atm‐6A minus ERA‐Interim. In (e) and (f), the mean root mean square (RMS) 20–90°N difference with ERA‐Interim is given on top right. In (d), the change of the root mean square difference with ERA‐Interim (ΔRMS) is indicated. In (d), the red contours provide the Atm‐5DL daily band‐pass DJFM 500‐hPa geopotential height standard deviation, in m. In (e) and (f), the red contours provide the ERA‐Interim daily band‐pass DJFM 500‐hPa geopotential height standard deviation, in m. Only grid points with statistical significance lower than 10% are colored.

Although changes in the direction and intensity of the climatological westerlies can have a large influence on the Arctic climate, a large fraction of the low‐troposphere transport of heat and moisture toward the Arctic is also related to the transient eddies. To measure how they are modified, we next evaluate the winter daily 500‐hPa geopotential height standard deviation, band‐pass filtered at 2.5–6 days (Blackmon, 1976). The geopotential height standard deviation of the model is quite realistic (Figures 3a–3c), with the Pacific and Atlantic storm tracks located at 50°N over both basins. Nevertheless, Atm‐6A and Atm‐5DL tend to slightly underestimate the variance over both storm tracks, while the variance is overestimated over land (Figures 3e and 3f), especially over northwestern America. In Atm‐6A, the variance is reduced almost everywhere around the globe in the polar and midlatitudes compared to Atm‐5DL (Figure 3d). The reduction of the overestimated variance over land explains the overall reduction of the 20–90°N RMSE from 4.72 m in Atm‐5DL to 3.81 m in Atm‐6A. The decreased variance in Atm‐6A is consistent with the weaker polar vortex described in Figure 2a if we assume that a weaker amplitude vortex is more stable.

**Figure 4:**
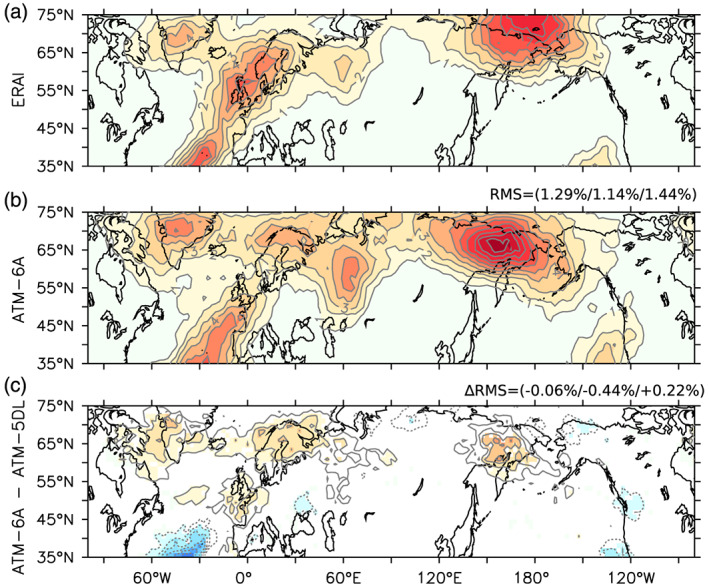
DJFM blocking frequency, in %, for (a) ERA‐Interim 1979–2014, (b) Atm‐6A, and (c) Atm‐6A minus Atm‐5DL. The contour interval is 1% for all panels. In (b), the mean root mean square (RMS) difference of ATM‐6A minus ERA‐Interim is given on top in three boxes (global 35–75°N/North Atlantic 100°E–40°W, 35–75°N/North Pacific‐Eurasia 60°W–120°E, 35–75°N). In (c), only grid points with statistical significance lower than 10% are colored. The change of the root mean square difference with ERA‐Interim (ΔRMS) for the same boxes as (b) is given on top.

To understand the impact on the midlatitude synoptic variability, we also investigate the blocking characteristics. The blockings are closely linked to the main mode of atmospheric variability (Davini et al., 2012; Woollings et al., 2008) and are usually not well represented in climate models, with underestimated blocking frequencies over Northern Europe (Davini & Cagnazzo, 2014). Pithan et al. (2016) attributed this underestimation to a lack of SSO drag in most models. A blocking index is defined following Scherrer et al. (2006), using the meridional gradient of daily geopotential height at 500 hPa and considering only blocking events lasting more than five consecutive days. When comparing with ERA‐Interim, the blocking frequency simulated by Atm‐6A is overestimated over the Urals and far eastern Siberia, while it is underestimated over the British Isles (see Figure 4). The SSO adjustment in Atm‐6A has however contributed to increasing the frequency of blocking over Greenland and Scandinavia that was largely underestimated in Atm‐5DL. From Atm‐5DL to Atm‐6A, the blocking frequency RMSE is reduced by 0.44% over the North Atlantic section (Figure 4). However, the blocking frequency has been degraded in far eastern Siberia, with an increased RMSE of 0.22%.

### 3.3 Zonal‐Mean Changes

Increasing orographic drag to cool the polar regions poses a challenge since, in the past, orographic gravity wave drags were often introduced to warm the upper troposphere and low stratosphere (Palmer et al., 1986). The arguments involve downward control principles (Haynes et al., 1991), where an upper‐level drag is balanced via the Coriolis torque by a poleward Transformed Eulerian Mean (TEM) meridional velocity (called v*) that corresponds to the upper branch of an indirect circulation cell. In the poleward branch of the cell and below where the drag is applied, the TEM vertical velocity (called w*) is downward (w* < 0) causing adiabatic warming. A key aspect of the downward control argument is that the vertical integration used to predict the meridional circulations starts at *z* = ∞ to use the boundary condition ρw* = 0. Integration from the surface is systematically disregarded (“upward control”) based on the argument that the surface frictions can easily adapt to enforce quasi‐steady states. In the case of the SSO modifications tested here, the surface drags are imposed in the lower troposphere, and the “downward control” argument is not easy to adapt. Seminal papers like Eliassen (1951) show that in principle, a drag applied near the surface can cause direct cells above where the drag is applied, which is a northward low‐level flow yielding by mass conservation an upward flow north and hence adiabatic cooling. According to past literature, one nevertheless needs to be extremely careful with such conclusions and test the changes in surface friction and upper‐level forcing by the resolved waves.

**Figure 5:**
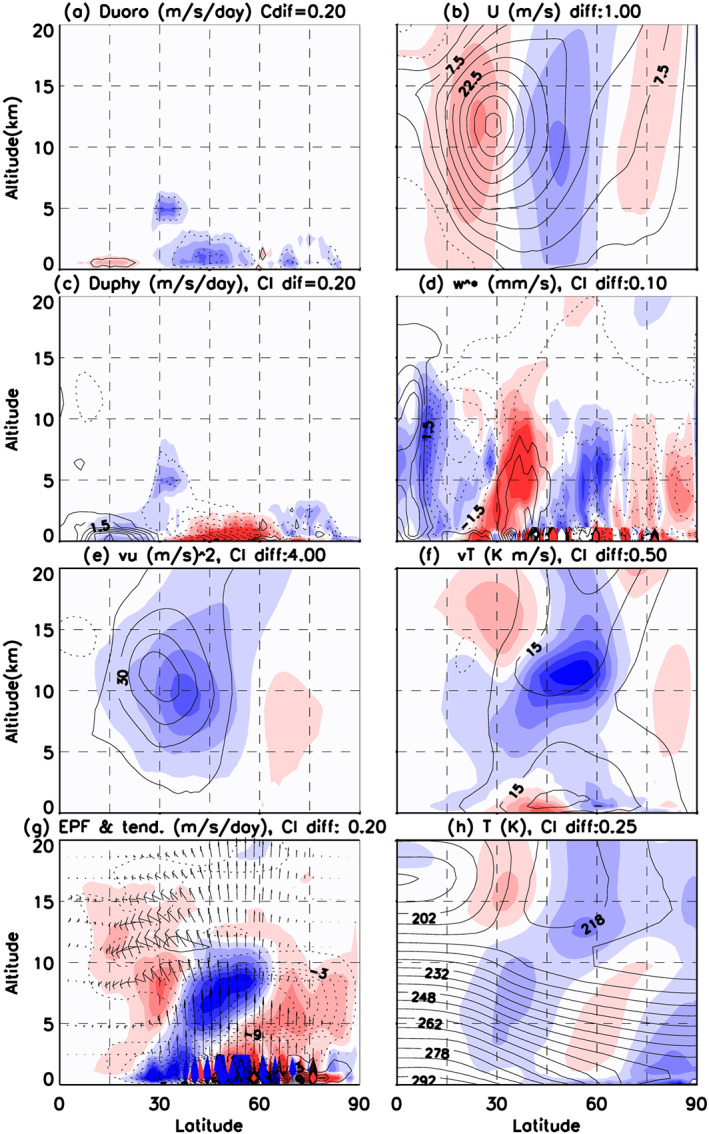
DJFM zonal‐mean circulation illustrated by (contour) the climatological fields in Atm‐6A and (color) difference of Atm‐6A minus Atm‐5DL (contour interval [CI] provided on top of each panel): (a) zonal‐mean zonal wind tendency due to orographic drag, in m s^−1^ day^−1^; (b) zonal‐mean zonal wind, in m s^−1^; (c) zonal‐mean zonal wind tendency due to atmospheric physics, in m s^−1^ day^−1^; (d) residual vertical velocity, in mm s^−1^; (e) eddy zonal wind flux, in m^2^ s^−2^; (f) eddy temperature meridional flux, in K m s^−1^; (g) zonal wind tendency implied by the Eliassen‐Palm flux divergence, in 10^2^ m s^−1^ day^−1^; and (h) zonal‐mean temperature, in K. In (g), the vectors show the climatological Eliassen‐Palm flux (vector, with a typical magnitude of 150 m^2^ s^−1^ day^−1^), using the scaling of Edmon et al. (1980).

Although the momentum budget equation can be used to interpret directly the changes of the mean meridional circulation resulting from changes in momentum fluxes, TEM quantities provide a more complete description of the atmospheric changes. To disentangle the feedbacks, Figure 5 presents zonal‐mean diagnostics of TEM quantities derived following Andrews et al. (1987). First in Figure 5a, one sees that the SSO drags in Atm‐6A and difference in SSO drags from Atm‐6A to Atm‐5DL are both negative at low levels in the Northern Hemisphere midlatitudes and polar regions as expected. The tendencies due to SSO lift are much weaker for the zonal flow (not shown). The zonal‐mean zonal wind in Figure 5b presents a subtropical jet with center around (28°N, 12 km) that is in agreement with observations. It is tilted poleward when altitude decreases, and the lower‐troposphere jet maximum (i.e., the eddy driven jet) is around 35–40°N. The impact of the changes in the zonal‐mean winds is consistent with Lott (1999), as the jet decreases above where the drag is applied, reducing the intensity of the eddy‐driven jet. Besides, the zonal wind increases in the subtropical regions shifting the subtropical jet equatorward. Importantly, the response to the changes in SSO drag is almost barotropic, consistent with the fact that the low‐level mountain drag is balanced by northward mass fluxes where it is applied, increasing the surface pressure northward and decreasing it southward. This is consistent with the changes in mass distribution due to mountains (Lott & D'andrea, 2005; Lott et al., 2004). The reduction in the baroclinic part of the jet, as indicated by the difference of zonal‐mean zonal wind between 300 hPa and 850 Pa at 35°N, is not significant at the 10% level (−0.46 m s^−1^).

The jet changes strongly impact the total drag in return. This is because above where the jet is decelerated the turbulent friction drag calculated by the boundary layer scheme is weaker (less negative) and vice versa. In our model, this more than balances the extra SSO drag between 30°N and 60°N where the total drag in Atm‐6A is weaker than in Atm‐5DL (Figure 5c). Interestingly, north of 65°N, the SSO drag is not balanced as much as elsewhere, and we suggest two reasons for this. The first is that the changes in the near‐surface winds are not as large as at lower latitudes; the second is that in these regions the near‐surface air is so stratified that the boundary layer does not develop well enough to efficiently balance the SSO drag.

To a certain extent, the TEM vertical velocity in Figure 5d responds to the near‐surface force in Figure 5c consistent with the case of Eliassen (1951) where drag is applied at the surface: North of 70°N, the residual vertical velocity is upward (w* > 0) above the surface and in the troposphere, consistent with the fact that the negative anomaly in low‐level drag is almost centered at 70°N and drives a direct cell aloft.

Nevertheless, as this interpretation challenges downward control principles, it is important to investigate the associated upper‐level changes in eddy forcing. In the classical “downward control” description of the meridional circulations, the meridional wind response to eddy‐driven forces is “supposedly” equilibrated by an opposing response due to the adjustment of the boundary layer. Such an equilibration is needed when one does long temporal average because the absence of equilibration yields a meridional transfer of mass and then a non‐stationary change in the zonal‐mean surface pressure field. As we adopt a more “upward controlled” view, one should test if our surface forces are in part compensated by changes in upper‐level eddy forces.

To some extent, we have begun to address this in Figure 3, where we found that the eddy activity was reduced in Atm‐6A. To evaluate this more precisely, Figures 5e and 5f show the zonal wind and temperature meridional fluxes due to the eddies, $\bar{v'u'}$ and $\bar{v'T'}$, respectively. We see that both decay in Atm‐6A compared to Atm‐5DL and also that near the surface between 50°N and 75°N, $\bar{v'T'}$ is smaller in Atm‐6A than in Atm‐5DL. This could well explain the polar cooling, with smaller meridional poleward heat flux decreasing the near‐surface temperature directly. What is also important, nevertheless, is the eddy forcing, which is the zonal wind tendency due to the divergence of the Eliassen‐Palm (EP) flux in Figure 5g. Note that in the upper troposphere, the EP fluxes converge and decelerate the zonal wind (Figure 5g, contours and vectors), while the EP fluxes diverge in the lower troposphere inducing the formation of the eddy‐driven jet. The difference in eddy forcing between Atm‐6A and Atm‐5DL is positive in the midtroposphere north of 50°N (Figure 5h, colors), so that the zonal wind is accelerated by eddies in this zone in Atm‐6A. If we equilibrate this positive difference in forcing by a negative difference in TEM meridional wind v*, according to downward control, it is associated with a direct anomaly in meridional circulation below, with reduced polar subsidence in Atm‐6A, and decreased near‐surface temperature north of 60°N.

### 3.4 Air Temperature Changes

**Figure 6:**
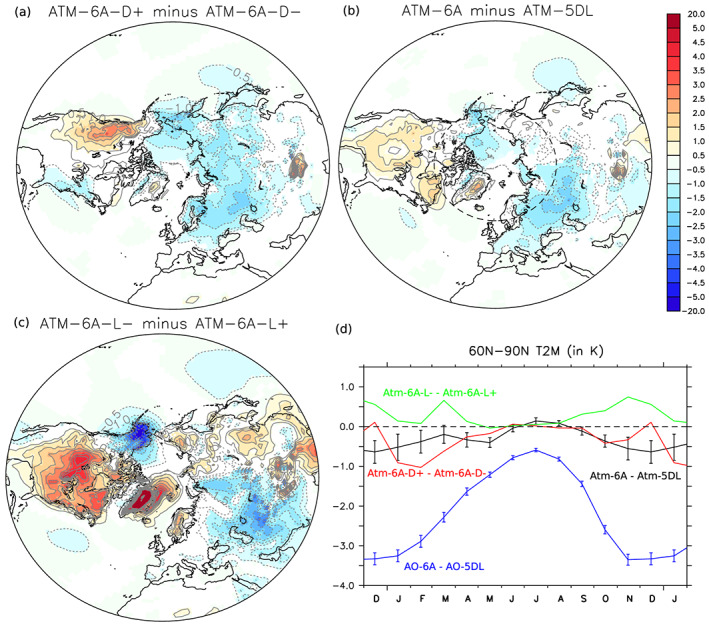
(a) DJFM 2‐m air temperature difference, in K, of Atm‐6A‐D+ minus Atm‐6A‐D−. Only grid points with statistical significance lower than 10% are colored. The latitude 60°N is shown with a dashed circle. (b) Same as (a), but for Atm‐6A minus Atm‐5DL. (c) Same as (a), but for Atm‐6A‐L− minus Atm‐6A‐L+. (d) Mean 2‐m air temperature changes over the polar cap (60–90°N) induced by SSO modifications; black: Atm‐6A minus Atm‐5DL; red: Atm‐6A‐Drg+ minus Atm‐6A‐Drg−; green: Atm‐6A‐Lft− minus Atm‐6A‐Lft+; blue: AO‐6A minus AO‐5DL. The error bars indicate the standard errors of the mean.

To evaluate how the upper air diagnostics translate in the boundary layer, Figure 6b shows the 2‐m temperature difference between Atm‐6A and Atm‐5DL. In Atm‐6A, North America is warmer, but most of the other regions are cooler, that is, Eurasia, and most importantly for sea ice, a large part of the Arctic. In the other sensitivity experiments, a similar warming, but with larger amplitude over North America, is reproduced for a large decrease of the lift (Figure 6c), as well as a cooling in western Eurasia. These surface temperature changes are consistent with the modified standing wave pattern (Figure 2f), with the anomalous southerly flow over North America and anomalous easterly flow in western Eurasia. However, as the lift is only slightly decreased in Atm‐5DL when compared to Atm‐6A (see Table 1), the effect of the lift is likely not dominant in Atm‐6A minus Atm‐5DL over Eurasia and the Arctic. The cooling simulated in Atm‐6A over the Arctic and Eurasia is somewhat similar to the one simulated when increasing the drag (Figure 6a, also given in Cheruy et al., 2020, their Figure 9). The standing wave pattern is only modified over America by the increasing drag, with the anomalous southerly (northerly) flow in eastern (western) America, thereby producing surface warming (cooling). The cooling produced over Eurasia and the Arctic is likely dominant in the zonal‐mean temperature changes illustrated previously (Figure 5h). As discussed in Cheruy et al. (2020), as the atmospheric model has a warm winter bias over the Northern Hemisphere midlatitude, the SSO changes in Atm‐6A partly reduce the bias over Eurasia but increase it over North America.

The Arctic cooling is occurring only during the winter in Atm‐6A (from November to March; Figure 6d, bottom, black curve) and is consistent with a dominant effect of the increasing drag (red curve), while little air temperature changes are simulated during the other seasons. Although in the TEM diagnostics we insisted on the role of the increased drag, the decreased lift (green curve) may also attenuate the dominant drag‐induced near‐surface cooling in March or in November. This again demonstrates the importance of the eddy forcing, the lift being important for the planetary waves.

## 4 Impacts in the Ocean‐Atmosphere Coupled System

### 4.1 Atmospheric Circulation Changes

**Figure 7:**
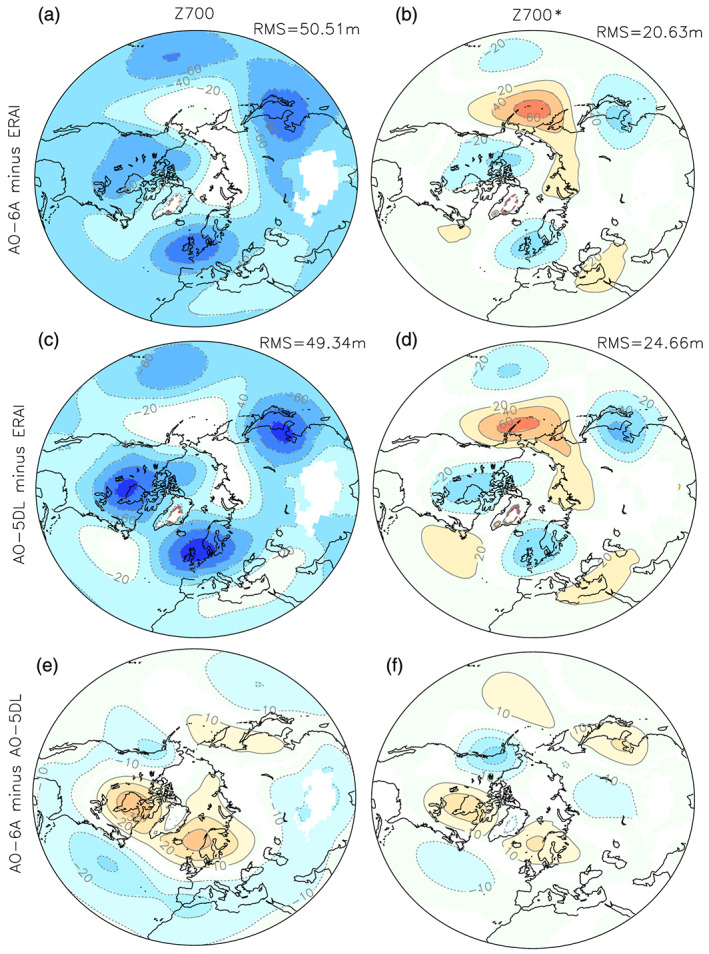
(a) Geopotential height at 700 hPa averaged over the winter months (DJFM) for AO‐6A minus ERA‐Interim and (b) its zonally asymmetric component. (c) and (d) are the same as (a) and (b), but for AO‐5DL minus ERA‐Interim. (e) and (f) are the same as (a) and (b), but for AO‐6A minus AO‐5DL. In (a)–(d), the root mean square (RMS) 20–90°N difference with ERA‐Interim is indicated on top. In all panels, only grid points with statistical significance lower than 10% are colored.

The planetary standing wave of the ocean‐atmosphere coupled experiments based on the atmospheric model component studied previously is shown in Figure 7. The overall biases of the 700‐hPa geopotential height in AO‐5DL resemble the biases illustrated previously in the atmosphere‐only experiments: a too deep polar depression and three anomalous troughs over north‐eastern America, northern Europe, and eastern Asia (see Figure 7c). The 700‐hPa geopotential height biases are larger in the coupled model (cf. Figures 1c and 7c), with a maximum bias of ~100 m in AO‐5DL and ~80 m in Atm‐5DL.

The 700‐hPa height changes (Figures 7e and 7f) in AO‐6A relative to AO‐5DL are qualitatively similar to that illustrated previously in the atmosphere‐only experiments (Figures 2a and 2b), with a strengthening of the geopotential height over the Arctic in AO‐6A when compared to AO‐5DL and a weakening over the 20–40°N latitude band, especially over the North Atlantic. When compared to AO‐5DL, two dominant ridges are simulated, one downstream of the Rockies over north‐eastern America and another one over northern Europe. A smaller ridge is also simulated in eastern Asia, downstream of the Tibetan Plateau. Furthermore, two troughs are simulated upstream of the two major Northern Hemisphere mountain ranges. As in stand‐alone atmospheric simulations, the SSO modification in AO‐6A alleviates the atmospheric circulation biases for the asymmetric component as compared to AO‐5DL (Figures 7c and 7d, RMSE from 24.7 to 20.6 m), but the response is weaker in the coupled model case, except over Northern Europe. For example, the same SSO modification (i.e., 6A minus 5DL) in the atmospheric experiment led to changes of up to 50 m over north‐eastern America (Figure 2a), while the changes are of the order of 30 m in the same region in the AOGCM experiments (Figure 7e). The resulting geopotential height in AO‐6A (Figure 7a) remains too strong over the Arctic compared to ERA‐Interim (Figure 1a) and too weak over the midlatitudes, yet less than in AO‐5DL (Figure 7e). The biases of the 700‐hPa geopotential height are nevertheless larger than AO‐5DL in AO‐6A (RMSE increase from 49.3 to 50.5 m) as the geopotential height decreases in the latitudinal band 20–40°N.

The difference in duration between the coupled and atmospheric experiments might explain the larger changes simulated in the 30‐yr atmosphere‐only experiments, as the internal variability is presumably better removed in the coupled experiment (duration ≥200 yr). Nevertheless, a comparison of the pairwise differences reveals that the changes are indeed significantly weaker in the coupled model experiments (Figure S2).

**Figure 8:**
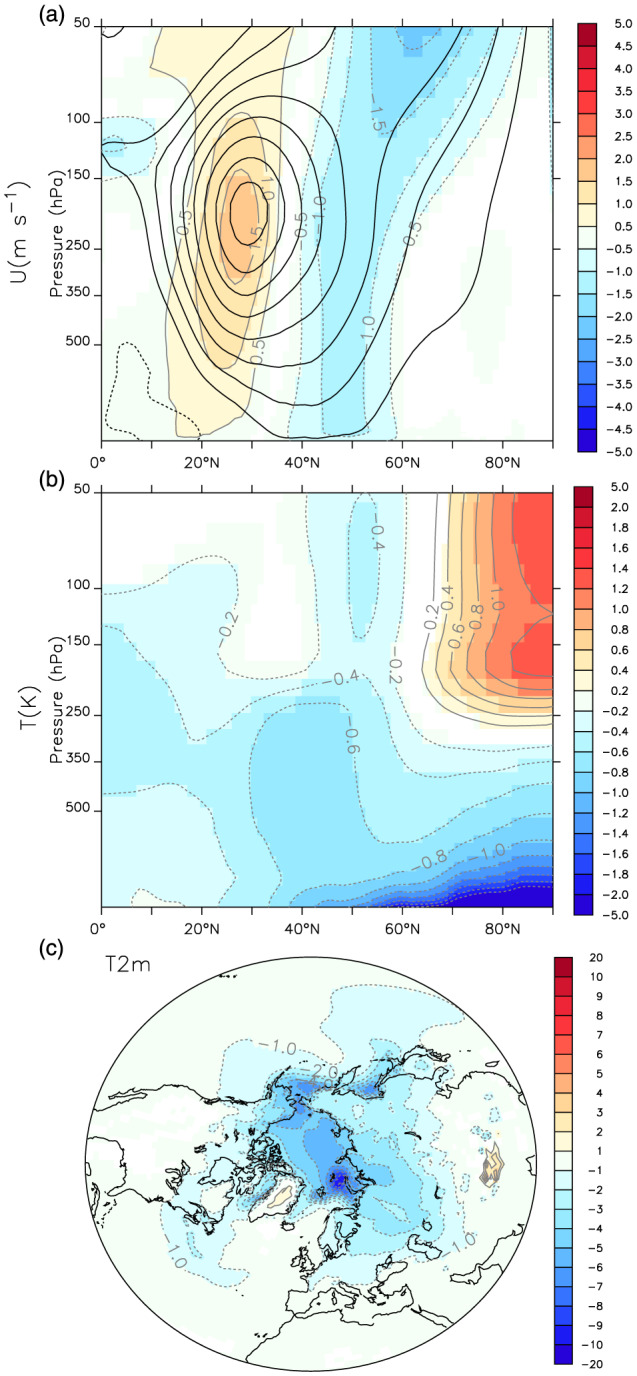
(a) DJFM difference of zonal‐mean zonal wind (in m s^−1^) of AO‐6A minus AO‐5DL. (b) Same as (a), but for the zonal‐mean temperature (in K). (c) DJFM 2‐m air temperature difference, in K, of AO‐6A minus AO‐5DL. In all panels, only grid points with statistical significance lower than 10% are colored.

The zonal‐mean zonal wind anomalies in AO‐6A relative to AO‐5DL, in the coupled simulations (Figure 8a), are also similar to that shown in the analogous atmosphere‐only simulations (Figure 5b). Both show a barotropic enhancement of the subtropical jet in its equatorward flank and a weakening of the eddy‐driven jet at 50°N. Nevertheless, consistently with the geopotential height response, the changes of AO‐6A minus AO‐5DL are about half of Atm‐6A minus Atm‐5DL. The associated zonal‐mean temperature changes are much larger in the coupled model (Figure 8b). Indeed, the lower‐troposphere cooling is quite intense, with a cooling of more than 2 K north of 60°N. A clear cooling is also simulated elsewhere in the troposphere, with values of −0.2 to −0.4 K in the tropics, and amplified values in the upper troposphere, as expected from the adjustment of the moist adiabat. On the other hand, warming is simulated in the polar stratosphere, and the stratospheric polar vortex weakens. The surface air temperature (Figure 8c) is about 3 K cooler over the whole Arctic, with a maximum cooling up to 8 K occurring over the Barents and Okhotsk Seas where the sea ice cover is thin and particularly sensitive to climate fluctuations. The cooling also extends over the Eurasian continent and, to a lesser extent, into the North Pacific and Atlantic.

The atmospheric variability in the coupled model also shows a decreasing 500‐hPa geopotential height variance similar to that of the atmosphere‐only simulations, but with weaker amplitude (Figure S3a). The blocking frequency also increases over Scandinavia (Figure S3b). Such an increase is larger than the one simulated in the atmospheric experiments (Figure 4), with a blocking frequency RMSE reduced by 0.77% over the North Atlantic. Over northern Siberia, the RMSE is almost unchanged. In the upper troposphere, the meridional zonal wind and temperature transports are also similar in the coupled model and the atmosphere‐only case (Figures S3c and S3d). However, the lower‐troposphere meridional temperature transport at 30–60°N increases in the coupled experiments as a result of the larger meridional temperature gradient. Nevertheless, the anomalous residual vertical velocity is still found to be ascending (negative) north of 60°N for the AOGCM case (Figure S3f), as the lower‐tropospheric lapse rate increases.

We conclude that in the coupled model the overall dynamical changes due to the SSO modification are similar to the ones inferred from the atmospheric model but weaker. However, these changes in the coupled model are superimposed onto a lower‐tropospheric cooling over the polar cap. The next subsection focuses on the associated sea ice extension and thickness.

### 4.2 Ocean and Arctic Sea Ice

**Figure 9:**
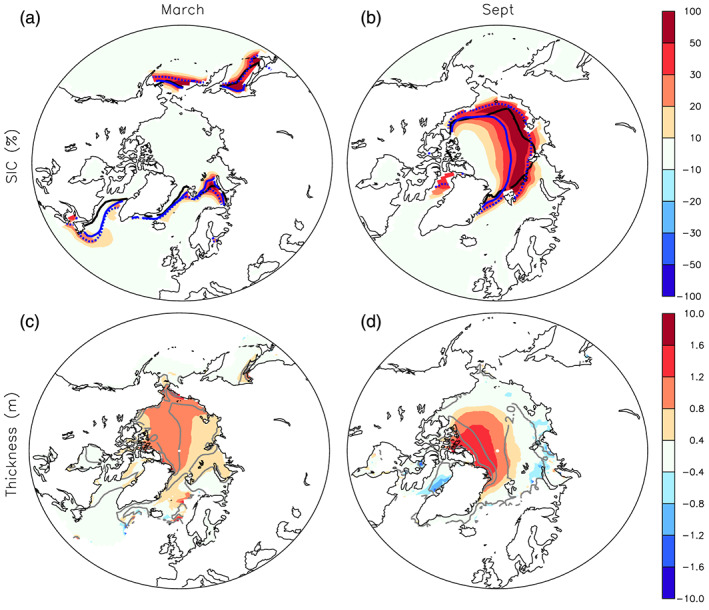
Arctic sea ice concentration, in %, in (a) March and (b) September. The black contour provides the observed sea ice concentration of 50% from 1979 to 2014. The blue contour illustrates the same contour for AO‐6A (dashed line) and AO‐5DL (full line). Arctic sea ice thickness, in m, in (c) March and (d) September. The gray contours give the mean value in AO‐5DL. In all panels, the color illustrates AO‐6A minus AO‐5DL.

The Arctic sea ice extent is increased in AO‐6A as compared to AO‐5DL in both summer and winter. In winter, the increase is mostly located over the Northern Pacific and the Barents Sea (Figure 9a), while the sea ice concentration decreases locally over the Labrador Sea. The Arctic sea ice thickness also shows a large increase of ~0.8 m in the central Arctic (Figure 9c): It is ~3 m in AO‐5DL, and it raises up to ~3.8 m in AO‐6A. In summer, the sea ice extent increases especially along the coast of Russia in the eastern Arctic (Figure 9b). The multiyear ice thickness also increases by about 1 m off Greenland (Figure 9d). Our interpretation is that the colder winter temperature induced by the modified SSO (see Figure 6a) has led to enhanced Arctic sea ice growth in the coupled model. The resulting larger sea ice volume can favor a colder Arctic with a larger summer sea ice extent, as found, for example, in model experiments designed to study the influence of sea ice initialization (Blanchard‐Wrigglesworth et al., 2011; Holland et al., 2011), or when assimilating sea ice thickness in models (Blockley & Peterson, 2018). Besides, the summer sea ice changes may be amplified by the sea ice‐albedo feedback. In summary, the impact of SSO modifications over the Arctic is largely modified by the ocean‐atmosphere coupling, leading to a larger thermodynamic response when compared to the atmosphere‐only model. As the sea ice insulates the ocean from the atmosphere, the more extended sea ice inhibits the heat release from the ocean to the atmosphere in winter, thereby reinforcing the winter cooling. This feedback explains the maximum cooling in November and December (see Figure 6d, blue line). The ice‐albedo feedback may contribute to the smaller summer cooling. Lastly, we note that the SSO modification has corrected the underestimated summer sea ice extent simulated present in AO‐5DL, as illustrated in Figures 9a and 9b by the observed and simulated 50% contour for the sea ice concentration.

**Figure 10:**
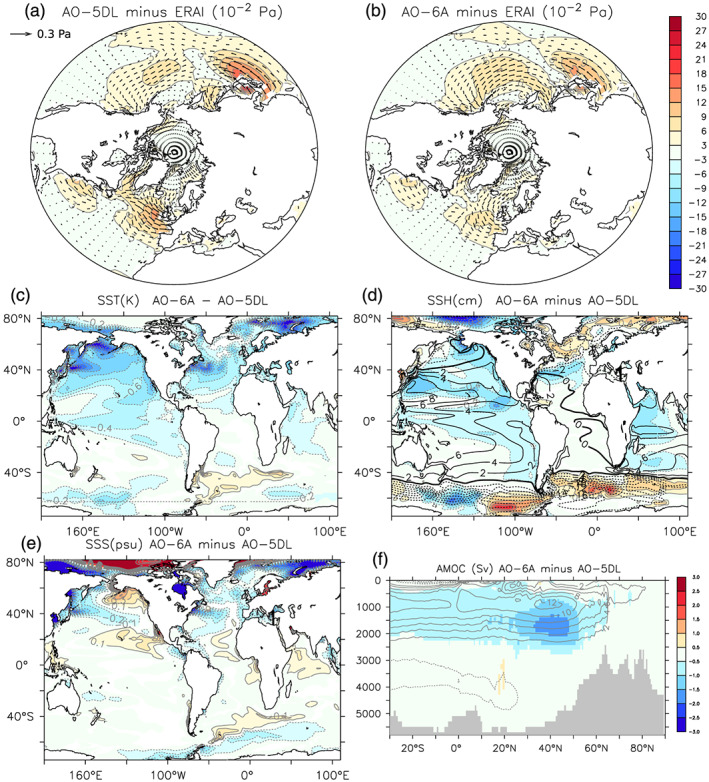
Annual mean wind stress difference between (a) AO‐6A and ERA‐Interim and (b) AO‐5DL and ERA‐Interim. The color indicates the magnitude of the difference, in 10^−2^ Pa^−1^, while the vectors indicate the difference in Pa. (c) Annual mean SST (contour interval = 0.2 K) difference of AO‐6A minus AO‐5DL. (d) Same as (c), but for the SSH (contour interval = 2 cm). The mean SSH (in cm) in AO‐6A is indicated in black contour. (e) Same as (c), but for the SSS (contour interval = 0.1 psu). (f) Yearly Atlantic meridional overturning stream function (in Sv) changes for AO‐6A minus AO‐5DL. The mean Atlantic meridional overturning stream function (in Sv) in AO‐6A is indicated in gray contour. In (c)–(e), only grid points with statistical significance lower than 10% are colored.

The oceanic changes are not restricted to the Arctic. The lower‐tropospheric westerlies are overestimated in AO‐5DL over the eastern North Atlantic and the Kuroshio extension in the Pacific (Figure 10a). The simulation AO‐6A (Figure 10b) shows a reduction of these two biases, even if the underestimation of the wind stress in the eastern Pacific becomes more pronounced. This reduction of the westerlies is associated with a southward shift of the Northern Hemisphere western boundary oceanic currents, namely, the Gulf Stream and Kuroshio. This can be seen through the maximum cooling located in the western Pacific and Atlantic at 40–45°N (Figure 10c). This is also consistent with the sea surface height (SSH) reduction at the same locations (Figure 10d). In AO‐6A minus AO‐5DL, the sea surface salinity is also reduced in the subpolar North Atlantic (Figure 10e), which is consistent with a decreasing northward salt transport related to the southward shift of the North Atlantic current. As discussed in Boucher et al. (2020), IPSL‐CM6A‐LR (identical to AO‐6A here) has an important cold (~3°C) and fresh (~1 psu) bias in the North Atlantic. It also shows a cold (~1°C) bias off Japan. As the anomalies indicated by AO‐6A minus AO‐5DL are smaller but consistent with such biases, SSO changes have contributed to amplify these biases. We also note that the warm bias in the Bering sea has been reduced by the SSO modification.

Cooling is also simulated in the equatorial Pacific and the Indian Ocean in AO‐6A as compared to AO‐5DL. It might be explained by the global response to increased sea ice cover. Many previous studies indeed found that sea ice loss causes a tropical warming in coupled models, called “a mini‐global warming” (Blackport & Kushner, 2017; Deser et al., 2014), by analogy with the warming induced by increasing greenhouse gases. Such a tropical impact is explained by the water vapor feedback and ocean circulation changes (Deser et al., 2016). The tropical cooling produced by the sea ice increase in our experiments is very comparable to the results in these previous studies, but with an opposite sign. We will illustrate next the changes in the meridional energy transports.

### 4.3 Meridional Energy Transport

In the coupled simulations, the atmospheric and oceanic meridional energy transports change as a response to the new surface and top‐of‐atmosphere energy budgets. The atmospheric and oceanic energy transports are calculated using the top of the atmosphere radiative budget and the net surface heat flux integrated from 90°S. As the energy non‐conservation is stationary (not shown), we remove the mean non‐conservation term before calculation.

**Figure 11:**
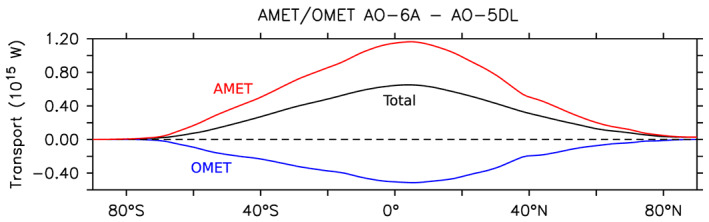
Total (black line), atmospheric (red line), and oceanic (blue line) annual mean meridional energy transport difference, in 10^15^ W, for AO‐6A minus AO‐5DL.

In the coupled experiments, the extension of Arctic sea ice in AO‐6A relative to AO‐5DL leads to a decrease of incoming shortwave radiation over the Arctic, caused by the increased surface albedo. This implies an increase of the total northward meridional energy transport, as illustrated in Figure 11 (black line). The atmospheric meridional energy transport (AMET; red line) accounts for most of this increase. The AMET increase is consistent with the lower‐tropospheric meridional temperature transport in midlatitudes (Figure S3c). In the tropics, the AMET changes are consistent with the Hadley cells modifications expected from the Arctic cooling (Yoshimori et al., 2018), with a direct anomalous cross‐equatorial cell. The anomalous cell leads to northward meridional geopotential transport in its upper branch and increasing southward heat and moisture transport in its lower branch (Figure S4). However, the northward oceanic meridional energy transport (OMET; blue line) is reduced, which damps the influence of the AMET increase. The OMET reduction is consistent with the weaker Atlantic meridional overturning stream function (Figure 10f). In AO‐6A minus AO‐5DL, the decreasing subpolar North Atlantic salinity (Figure 10e) weakens the seawater density in the subpolar gyre, which likely leads in turn to the AMOC weakening.

## 5 Discussions and Conclusion

During the tuning of the IPSL‐CM6A‐LR model, the parametrized orography was modified to alleviate the biases of the atmospheric circulation resulting from the updated model physics. We increased the orographic lower‐tropospheric blocking effect (so‐called drag). We also decreased the lift, which is a force perpendicular to the local flow. The lift was designed to represent the dynamical separation from the large‐scale flow of the air in narrow valleys (Lott, 1999). The SSO changes implemented cause a reduced polar depression, as well as a better simulation of the Northern Hemisphere stationary wave pattern. Furthermore, we noticed a lower‐tropospheric cooling at 60–90°N over the Arctic. These changes are mainly due to the increased lower‐tropospheric drag. This effect is counter‐intuitive, as previous works found that enhanced drag generally warms the midlatitudes and polar regions (Palmer et al., 1986). Using TEM diagnostics in atmosphere‐only experiments, we showed that the cooling is driven by the weaker eddy activity, which decreases the northward heat and momentum transport. In the coupled model, the same SSO modification is found to have a large impact on Arctic sea ice, as the lower‐tropospheric atmospheric cooling is amplified by the winter sea ice growth and a reduced oceanic heat loss. Nevertheless, the changes in the standing wave or zonal winds are weaker than in the atmosphere‐only experiments.

The adjustment of the SSO parameterization in IPSL‐CM6A‐LR has therefore contributed to restoring the Arctic sea ice cover, which was initially too sparse. In our case, the Arctic sea ice bias was associated with a warm winter air temperature bias, which was thus also reduced. Nevertheless, several other negative impacts are also found, so that caution is needed before applying such SSO modifications. In particular, increasing the SSO drag and decreasing the lift have led to a reduction of the AMOC, which is rather weak in this model (about 13 Sv; Boucher et al., 2020). We suggest that the AMOC changes are here induced by the weaker westerlies in the Eastern Atlantic, shifting southward the North Atlantic current, and decreasing the salinity transport toward the subpolar gyre. The wind‐induced southward shift of the North Atlantic current has also degraded the cold and fresh bias present in the central Atlantic (Boucher et al., 2020). This bias is a common feature in many models using a low‐resolution ocean.

The surface air temperature impact was also specifically investigated outside the Arctic. In the atmospheric simulations, the SSO modification is found to modulate the contrast of air temperature between North America and Eurasia. This reflects the influence of SSO drag on the planetary stationary wave. The SSO drag also cools Eurasia, as it weakens the air advection from the warm Atlantic toward the land. The air temperature modification is also partly caused by the lift, which directly modifies the Northern Hemisphere standing wave pattern.

The results shown are likely sensitive to the model. However, the CMIP5 models all have biases of the North Atlantic storm track and European blockings. These biases were found to be quite similar to those produced in a simulation with a deactivated low‐level orographic blocking effect (Pithan et al., 2016). Furthermore, the low‐level winter warm biases over Arctic sea ice are also common to many other models (Graham et al., 2019). This suggests that a deficit of low‐level drag is also present in other climate models, and more work might therefore be needed to understand the implications for Arctic sea ice and the oceanic circulation biases in the other AOGCMs.

## Supporting information

Supporting Information S1

## Data Availability

The data of the sensitivity experiments supporting the conclusions of the study can be obtained online (https://doi.org/10.5281/zenodo.3714902).
