# Supplementary material for: Alleviation of an Arctic Sea Ice Bias in a Coupled Model Through Modifications in the Subgrid‐Scale Orographic Parameterization
Source: J Adv Model Earth Syst. 2020 Sep 21;12(9):e2020MS002111. doi: 10.1029/2020MS002111 (PMC7540048; doi:10.1029/2020MS002111)
Supplement: Supplementary file 1 — Supporting Information S1 [file JAME-12-e2020MS002111-s001.pdf]

Journal of Advances in Modeling Earth System

Supporting Information for

Alleviation of an Arctic Sea Ice Bias in a Coupled Model through Modifications in the Subgrid-scale Orographic Parameterization

Guillaume Gastineau*[1], François Lott[2] , Juliette Mignot[1] and Frederic Hourdin[2]

[1]UMR LOCEAN, Sorbonne Université/IRD/MNHN/CNRS, IPSL, Paris, France.

[2]UMR LMD, ENS/Sorbonne Université/CNRS/Ecole polytechnique, Paris, France

Contents of this file

Figures S1 to S4

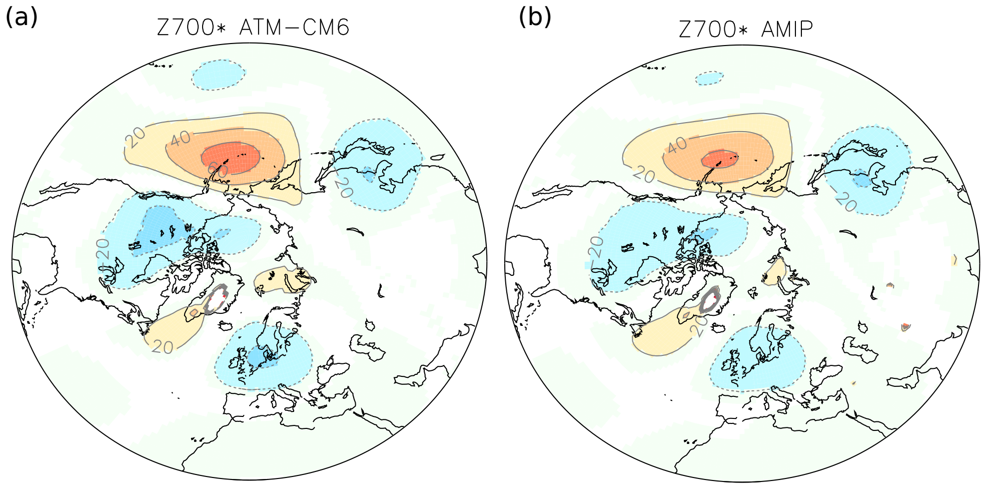

Figure S1. (a) Zonally asymmetric component of the geopotential height at 700-hPa, in m, averaged over the winter months (DJFM) for the Atm-6A minus ERA-Interim (1979-2014) difference (identical to Fig. 1f). (b) Same as (a), but for the ensemble mean of the 10 IPSL-CM6A AMIP CMIP6 simulations using interannual forcing over the 1979-2014 period. Only grid points with statistical significance lower than 10% are colored.

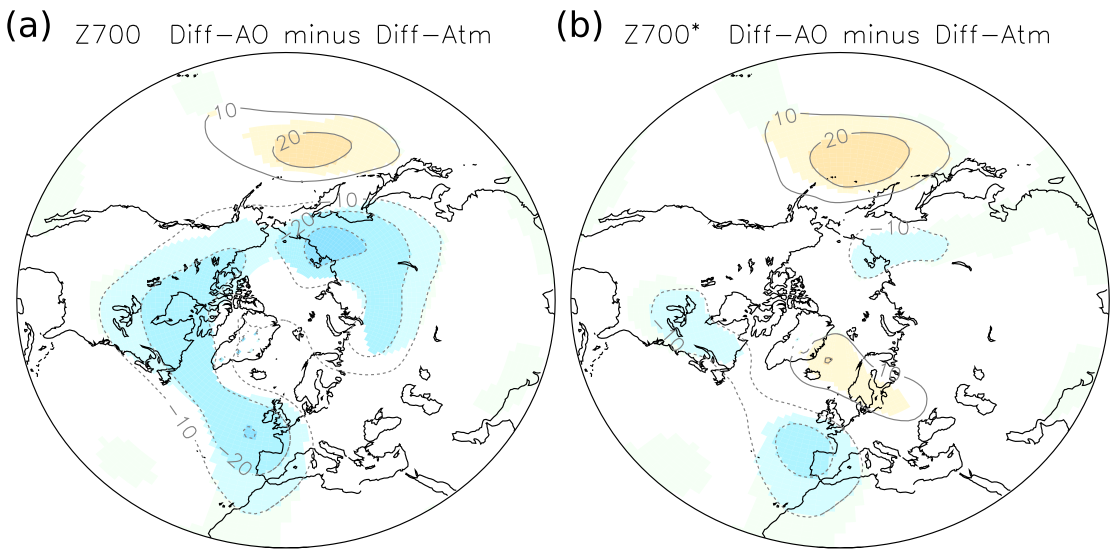

Figure S2. (a) Difference of the geopotential height at 700-hPa changes, in m, simulated in AO-6A minus AO-5DL compared to the changes simulated in Atm-6A minus Atm-5DL. (b) Same as (a), but for its zonally asymmetric component. The mean difference of AO-6A minus AO-5DL and its standard deviation are calculated pairwise over non-overlapping 40-yr periods using corresponding initial conditions, removing the first 30-yr and the last 10-yr of the five AO-5DL members. The mean and standard deviation for the difference between Atm-6A and Atm-5DL are also estimated pairwise. Only grid points with statistical significance lower than 10% are colored, as given by a Student *t*-test of the differences.

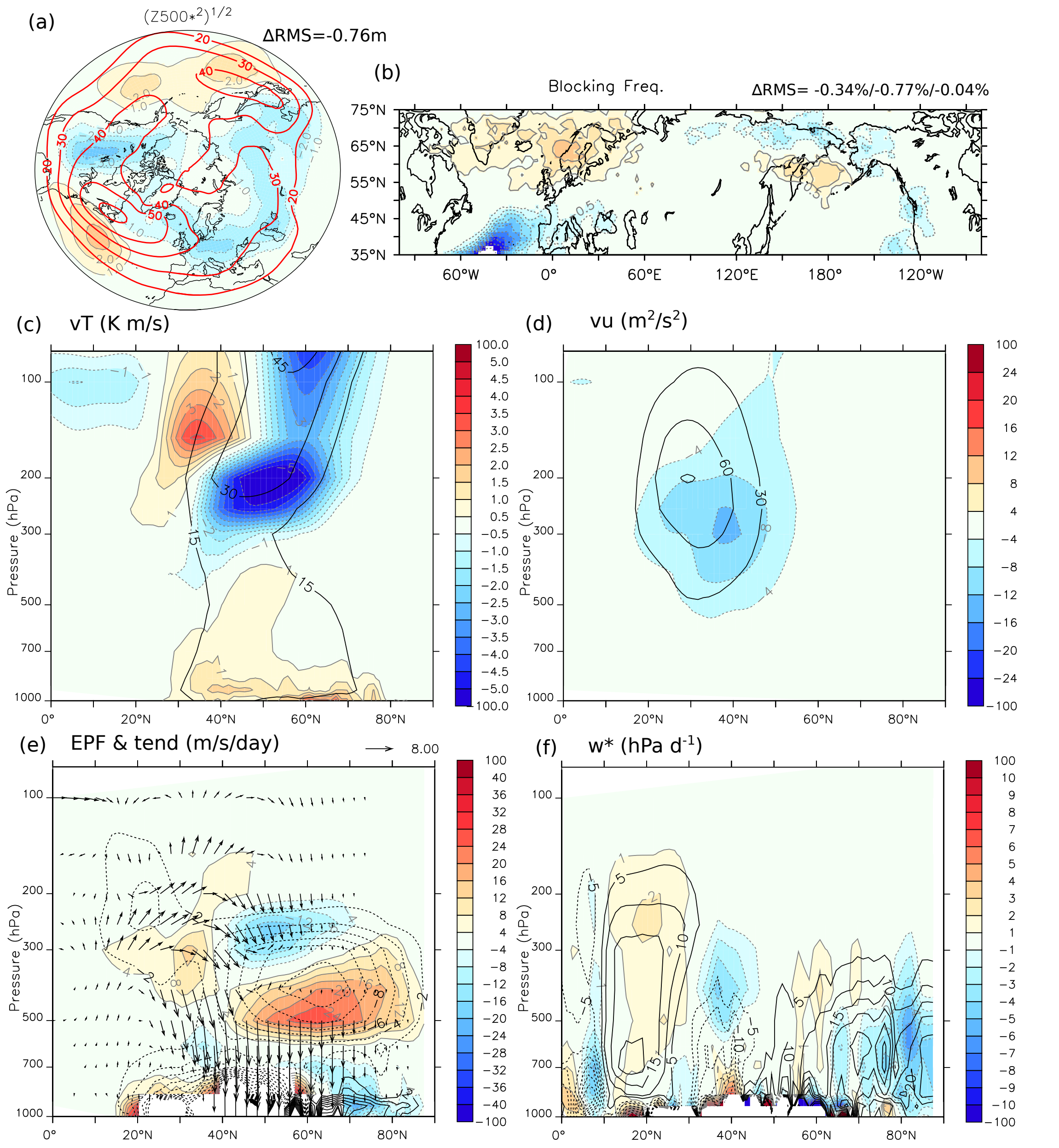

Figure S3. Characteristics of the DJFM circulation illustrated by (contour) the climatological fields in AO-6A and (color) AO-6A minus AO-5DL difference. (a) Daily band-pass (2.5-6 days) DJFM 500-hPa geopotential height standard deviation, in m. The red contours provide the daily band-pass DJFM 500-hPa geopotential height standard deviation, in m, for A0-5DL. The change of the 20°N-90°N root mean squared difference with ERA-Interim (ΔRMS) is given on top. (b) DJFM blocking frequency, in %, for AO-6A minus AO-5DL. The contour interval is 0.5%. The change of the root mean squared difference with ERA-Interim (ΔRMS) for three boxes (global 35°N-75°N/North Atlantic 100E-40°W 35°N-75°N/North Pacific-Eurasia 60°W-120°E 35°N-75°N) is given on top. (c) Eddy temperature meridonal flux, in K m s^{-1}. (d) Eddy zonal wind flux, in m^2 s^{-2}. (e) Zonal wind tendency implied by the Eliassen-Palm flux divergence, in 10^2 m s^{-1} d^{-1}; the vectors show the Eliassen-Palm flux (vector, in m^2 s^{-1} d^{-1}) difference of AO-6A minus AO-5DL, using the scaling of Edmon et al. (1980). (f) Residual vertical pressure velocity, in hPa d^{-1}.

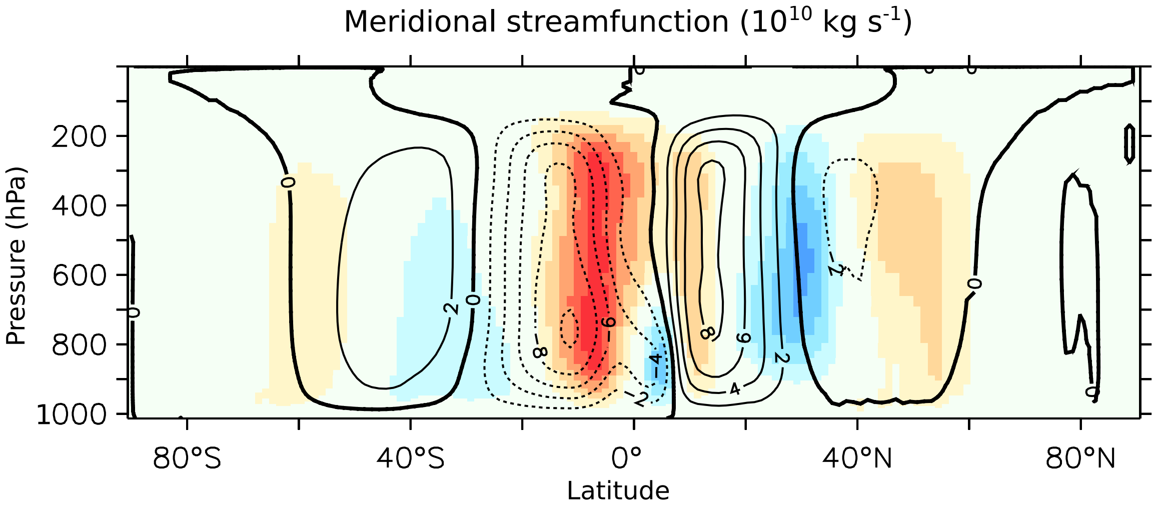

Figure S4. Annual mean meridional streamfunction, in 10^{10} kg s^{-1}, for (color; contour interval 0.1 10^{10} kg s^{-1}) AO-6A minus AO-5DL and (contour) AO-6A.
